# Short-Term Pilot Study to Evaluate the Impact of Salbi Educa Nutrition App in Macronutrients Intake and Adherence to the Mediterranean Diet: Randomized Controlled Trial

**DOI:** 10.3390/nu14102061

**Published:** 2022-05-14

**Authors:** Marina Gonzalez-Ramirez, Rocio Sanchez-Carrera, Angela Cejudo-Lopez, Mauricio Lozano-Navarrete, Elena Salamero Sánchez-Gabriel, M. Alfonso Torres-Bengoa, Manuel Segura-Balbuena, Maria J. Sanchez-Cordero, Mercedes Barroso-Vazquez, Francisco J. Perez-Barba, Ana M. Troncoso, M. Carmen Garcia-Parrilla, Ana B. Cerezo

**Affiliations:** 1 Departamento de Nutrición y Bromatología, Toxicología y Medicina Legal, Facultad de Farmacia, Universidad de Sevilla, 41012 Sevilla, Spain; marinagonzalez@us.es (M.G.-R.); rocsancar3@us.es (R.S.-C.); amtroncoso@us.es (A.M.T.); mcparrilla@us.es (M.C.G.-P.); 2 Fundación Pública Andaluza para la Gestión de la Investigación en Salud de Sevilla (FISEVI), Hospital Universitario Virgen Macarena, 41013 Sevilla, Spain; 3 Centro de Salud Bellavista, Distrito Sanitario de Atención Primaria Sevilla, 41013 Sevilla, Spain; angela.cejudo.sspa@juntadeandalucia.es (A.C.-L.); franciscoj.perez.barba.sspa@juntadeandalucia.es (F.J.P.-B.); 4 UGC Salud Pública Sevilla, Distrito Sanitario de Atención Primaria Sevilla, 41013 Sevilla, Spain; mauricio.lozano.sspa@juntadeandalucia.es; 5 Centro de Salud Puerta Este “Dr. Pedro Vallina”, Distrito Sanitario de Atención Primaria Sevilla, 41020 Sevilla, Spain; gelen.salamero.sspa@juntadeandalucia.es (E.S.S.-G.); malfonso.torres.sspa@juntadeandalucia.es (M.A.T.-B.); 6 Centro de Salud Esperanza Macarena, Distrito Sanitario de Atención Primaria Sevilla, 41003 Sevilla, Spain; manuel.segura.balbuena.sspa@juntadeandalucia.es; 7 Centro de Salud Los Bermejales, Distrito Sanitario de Atención Primaria Sevilla, 41013 Sevilla, Spain; mj.sanchez.cordero.sspa@juntadeandalucia.es (M.J.S.-C.); mercedes.barroso.sspa@juntadeandalucia.es (M.B.-V.)

**Keywords:** SAlBi educa, nutrition app, mHealth, Mediterranean diet, COVID-19, carbohydrate intake, fat intake

## Abstract

Promoting a healthy diet is a relevant strategy for preventing non-communicable diseases. This study aims to evaluate the impact of an innovative tool, the SAlBi educa nutrition app, in primary healthcare dietary counseling to improve dietary profiles as well as adherence to the Mediterranean diet. A multi-center randomized control trial comprising 104 participants was performed. Both control (*n* = 49) and intervention (*n* = 55) groups attended four once-weekly sessions focusing on healthy eating habits and physical activity, over one month. As well as attending the meetings, the intervention group used the app, which provides self-monitoring and tailored dietary advice based on the Mediterranean diet model. In a second intervention (one arm trial), the potential of SAlBi educa was evaluated for three months during the COVID-19 pandemic. At 4 weeks, the intervention group had significantly increased their carbohydrate intake (7.7% (95% CI: 0.16 to 15.2)) and decreased their total fat intake (−5.7% (95% CI: −10.4 to −1.15)) compared to the control group. Significant differences were also found for carbohydrates (3.5% (95% CI: −1.0 to 5.8)), total fats (−5.9% (95% CI: −8.9 to −3.0)), fruits and vegetables (266.3 g/day (95% CI: 130.0 to 402.6)), legumes (7.7g/day (95% CI: 0.2 to 15.1)), starchy foods (36.4 g/day (95% CI: 1.1 to 71.7)), red meat (−17.5 g/day (95% CI: −34.0 to −1.1)), and processed meat (−6.6 g/day (95% CI: −13.1 to −0.1)) intakes during the COVID-19 pandemic. SAlBi educa is a useful tool to support nutrition counseling in primary healthcare, including in special situations such as the COVID-19 pandemic. Trial registration: ISRCTN57186362.

## 1. Introduction

The latest World Health Organization (WHO) data reveal that, worldwide, 39% of adults are overweight and 13% are obese [1]. It is well known that overweight and obesity are major risk factors for non-communicable diseases (NCDs) such as cardiovascular diseases, type II diabetes, and musculoskeletal disorders, among others [1]. Therefore, efforts to prevent overweight and obesity through the promotion of a healthy diet and physical activity are important strategies for preventing such NCDs. 

The WHO states that, at the societal level, it is important to support individuals to make healthy food choices [1]. Effective actions by governments to create a healthy food environment include nutrition and dietary counseling by professionals at primary healthcare facilities [2]. In fact, communication between healthcare professionals and citizens is one of the main sources of information and training when addressing nutrition and physical activity. Traditionally, dietary counseling in primary healthcare consisted of both group sessions and individual advice, including recommendations for following a healthy diet using printed leaflets and guides [3]. Individual advice sessions last between 5 and 20 min during medical consultations [3,4,5]. Group sessions include four or five planned visits over a one- to twelve-month period, with each session lasting between 30 and 120 min. Sessions consist of motivational counseling, assistance, and educational activities [6,7,8,9]. Dietary counseling aims to disseminate knowledge, as well as encourage participants to follow a healthy diet [3]. Maderuelo-Fernandez et al. [10] showed that these interventions in five different countries, Holland, Italy, Spain, the United Kingdom, and the USA, moderately improved dietary habits, decreasing the consumption of saturated fats and increasing that of fruit, vegetables, and fiber, as well as increasing physical activity. The multi-center PREDIMED study conducted on the Spanish population increased adherence to the Mediterranean-Type Diet [11]. Despite all efforts, however, overweight and obesity are still on the rise [12].

The development of new technologies has raised health expectations, making the development of mobile health (mHealth) possible in order to provide efficient medical care everywhere and at all times [13]. The WHO Global Observatory for eHealth defines mHealth as medical and public health practice supported by mobile devices, such as mobile phones, patient-monitoring devices (e.g., heart rate monitor), personal digital assistants (PDAs), and other wireless devices [14]. New innovative tools for promoting healthy dietary habits, such as nutrition apps, are gaining popularity [15,16,17]. Several studies have demonstrated that nutrition apps are effective for weight loss (2–6.5 kg, between 3 to 6 months) in populations with overweight and diabetes [18,19,20,21] as well as for increasing fruit and vegetable intake (1–1.74 servings/day for two and three months) among the general and overweight population [22,23]. Few studies have focused on evaluating nutrition apps’ effects on changes in the dietary profile (macronutrients), showing an increase in carbohydrate intake (1.1%) and a decrease in fat intake (1.1%) in the general population [24]. 

mHealth use in primary healthcare dietary counseling appears to be a promising tool for implementing the transition towards healthier eating behavior. SAlBi educa is a pilot project comprising the design, development, and evaluation of a tailored nutrition application [25]. It aims to implement a nutritional education app in primary care nutrition counseling for the first time in the Andalusian public health system (South of Spain). The Andalusian Regional Ministry of Health, in collaboration with WHO, is one of the leading institutions in the European mHealth Innovation and Knowledge Hub, which aims to produce Knowledge tools for health systems and services on NCDs [26]. The SAlBi educa app offers personalized dietary counseling based on the Mediterranean diet model, since this model is one of the references for a healthy diet [27,28,29]. SAlBi educa was developed by health and nutrition professionals and is based on scientific evidence and nutritional recommendations from the WHO and the European Food Safety Authority (EFSA), and its usability was tested [25]. The app includes dietary self-monitoring (energy, total fat, carbohydrate, free sugar, proteins, and fiber), general and tailored feedback messages, and also suggests recipes and dishes typical of the Mediterranean model in order to achieve a balanced diet. At the moment, the app is aimed at Spanish speakers, although the basic methodology could be extended to other cultures.

The dietary intake of the Spanish population has changed significantly over the last 50 years, moving progressively away from the Mediterranean diet [30,31]. More recently, several studies have shown that the COVID-19 pandemic has also modified dietary habits [32,33,34,35,36,37,38]. Fish consumption has decreased while the consumption of dairy, sandwiches, and sweets, as well as total food intake and the number of meals and snacks, have increased. Therefore, this study aims to evaluate the potential of the SAlBi educa nutrition app to improve dietary profile (macronutrients), users’ adherence to the Mediterranean diet combined with dietary counseling in primary healthcare, as well as during special situations such as the COVID-19 pandemic.

## 2. Materials and Methods

### 2.1. Dietary Counseling + SAlBi Educa: Randomized Controlled Trial Study

#### 2.1.1. Design

A multi-center randomized controlled clinical trial with two parallel groups, the control, and the intervention group, was conducted. The trial was carried out in four primary healthcare centers in Seville (South Spain) belonging to the Andalusian Regional Public Healthcare Service. The intervention period lasted 4 weeks between January and February 2020. The study was previously registered as a clinical trial (Trial ID: ISRCTN57186362).

#### 2.1.2. Participants

Participants were recruited among the patients of the four participating healthcare centers. The specific exclusion criteria were as follows: (a) People whose physical or mental state made it impossible for them to complete the questionnaires and use the application; (b) people with a language other than Spanish that made it impossible for them to use the application and to understand the questionnaires correctly; (c) people under 18 years old, (d) people who attend to a monitored dieting program, or who used other nutrition apps, and (e) people who do not have a smartphone on which to install and use the application. 

#### 2.1.3. Randomization

**Figure 1:**
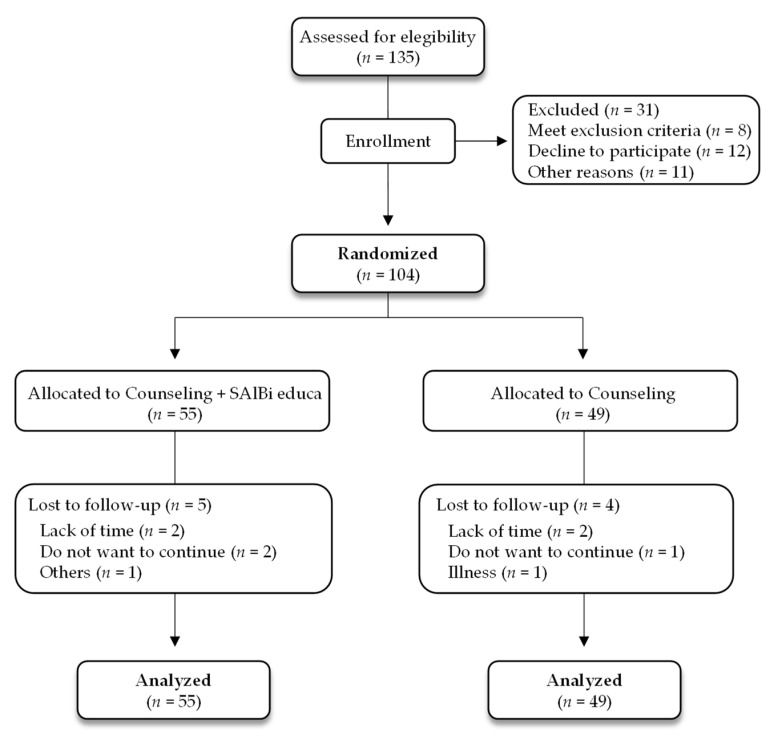
Study flowchart.

A total of 135 male and female participants were recruited, of whom 31 individuals were excluded (8 met exclusion criteria, 12 declined to participate, and 11 were excluded for other reasons). The sample subjects were randomized into two groups: (i) The control group (CG), who only received dietary counseling, comprising 49 individuals, and (ii) the intervention group (IG), who received dietary counseling + the app, comprising 55 individuals (Figure 1). A standardized program (https://www.randomizer.org/, accessed on 7 January 2020) was used by an independent researcher to generate the randomization sequence, and the data were blinded to the researcher performing the analysis for coding. The volunteers could not be blinded to the intervention due to the nature of the study.

#### 2.1.4. Common Intervention for Both Groups

The intervention took place during the standard advanced counseling on healthy eating habits performed in primary healthcare centers and following the “Guidelines on healthy habits in primary healthcare” [3]. The counseling consisted of planned weekly sessions, each lasting between 60 and 90 min over four consecutive weeks (January and February 2020), which focused on (i) basic concepts of the Mediterranean diet, (ii) nutrients and foods, (iii) physical activity, and (iv) purchasing food and understanding labeling (Figure S1 of Supplementary Material). Healthcare nutrition professionals with extensive experience moderated the sessions. Each session, at the end of which a printed summary was given to each volunteer, included a dynamic and didactic presentation of each topic in which volunteers and professionals could interact and set out questions. Since the objective of this study was to demonstrate the efficiency of the SAlBi educa app as a tool supporting primary healthcare dietary counseling, both groups (IG and CG) followed the four counseling sessions and only the IG used the app. The intervention was conducted in four public healthcare centers in Sevilla.

#### 2.1.5. Specific Intervention (Counseling + SAlBi Educa)

**Figure 2:**
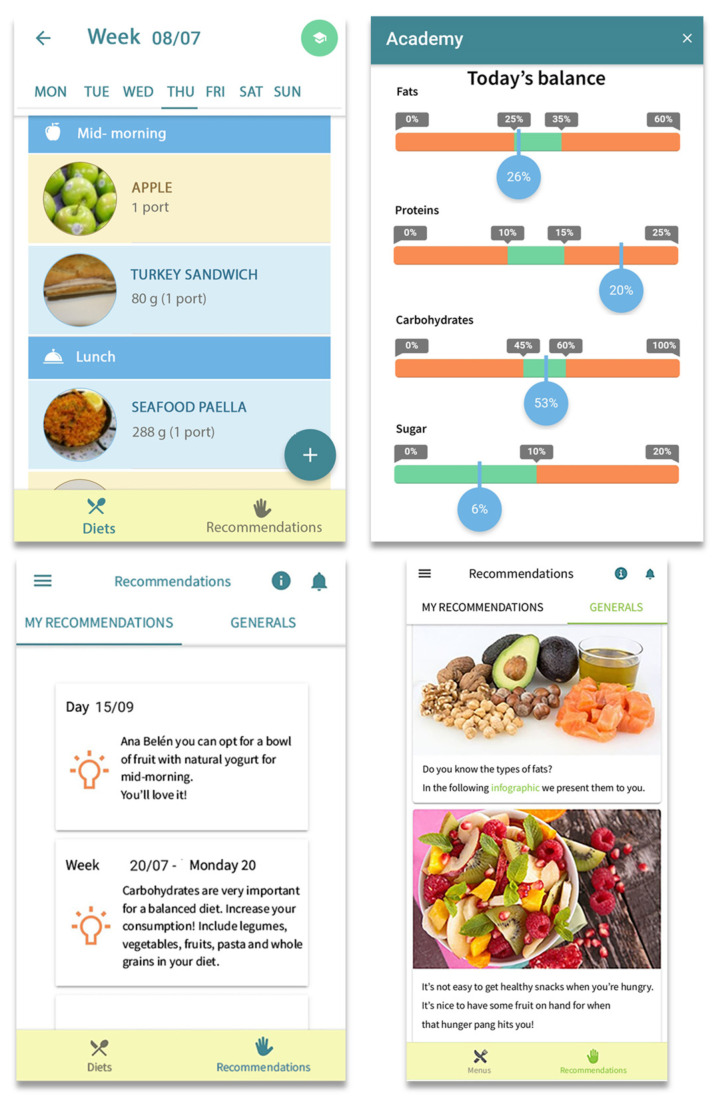
Screenshots of the SAlBi educa app.

During the first session, the IG received training in how to use SAlBi educa and was then authorized to use it. Volunteers were required to enter their daily intake in the application menu using its food and dishes database. SAlBi educa was developed by healthcare professionals from Seville Clinical Units (Family Doctors, Nurses, and Public Health and Health Promotion Technicians), professors and lecturers from the Nutrition and Food Sciences Department at the University of Seville, and potential users. The app was then tested for usability [25]. SAlBi educa offers personalized dietary counseling based on the Mediterranean diet model, estimating users’ energy requirements based on his/her data (sex, age, weight, height, and physical activity level). The application enables users to self-record their daily diet by selecting the foods and traditional Mediterranean recipes from the app database and indicate portion size. The nutritional information of foods and dishes was collected from the Spanish Food Composition Database (BEDCA) [39]. SAlBi educa includes nutrients (carbohydrates, proteins, total fats, free sugar, and fiber) and energy self-monitoring graphs (Figure 2). In addition, the app delivers (i) tailored feedback messages regarding the user’s food intake including recommendations on how to balance the diet, (ii) general nutrition messages, and (iii) infographics regarding basic concepts, the Mediterranean diet, food groups, and physical activity exercises, among others. All the data from the application were collected and analyzed. 

#### 2.1.6. Measurements

Data were collected via the following validated self-administered questionnaires: (i) Sociodemographic information, including age, sex, employment situation, and educational level, (ii) Mediterranean Diet (MedDiet) adherence [40], (iii) general nutrition knowledge [41], and (iv) dietary records to obtain the dietary intake (energy consumption, carbohydrates, proteins, free sugar, total fat, fiber, and food groups). 

The dietary record consisted of a structured form including the different meals (breakfast, mid-morning snack, lunch, afternoon snack, and dinner) [42,43]. Participants were trained and asked to include the ingredients of meals and the weight of portions, and record at least three days including one non-working day. Free sugars were estimated following the WHO definition as being all sugars added to foods or drinks by the manufacturer, cook, or consumer, as well as the sugars naturally present in honey, syrups, fruit juices, and fruit juice concentrates [2]. The fruit and vegetable estimation excluded potatoes, sweet potatoes, cassava, and other starchy roots following the WHO description [2]. The MedDiet adherence questionnaire assesses dietary habits associated with the Mediterranean diet (use of olive oil, consumption of fish, legumes, etc.). Scores over eight are established for good adherence. The abovementioned questionnaires were completed at the beginning (before counseling and authorization to use the app) to gather baseline data and again at the end of the intervention. Additionally, the dietary record was also collected every week over the four weeks. Dietary intake in the intervention group was obtained from the app and automatically transformed into macronutrients, fiber, as well as energy.

Anthropometric measurements (height and weight) were taken by health professionals during the visits at the beginning and at the end of the study. Both the app and the rest of the materials used were provided in Spanish.

### 2.2. Evaluation of SAlBi Educa Effectiveness during the COVID-19 Pandemic: Online Trial Study

A new trial was set up to evaluate the effectiveness of SAlBi educa during the COVID-19 pandemic. Due to attendance restrictions at healthcare centers, this trial included one group that used the app SAlBi educa for three months between September 2020 and July 2021. Participants were recruited through the project’s social networks, emails, dissemination at the University, the Seville Board of Education, and through the four primary healthcare centers mentioned in the previous study. The exclusion criteria were the same that those used in the previously described randomized controlled trial. A total of 125 volunteers were recruited, of whom 85 individuals were excluded (15 met exclusion criteria, 30 declined to participate, and 40 were excluded for other reasons). All the participants included in the study attended an initial online workshop during which they were trained in the use of the SAlBi educa app. Data were collected using the same self-administered questionnaires as in the randomized control trial, but the forms were completed online both at baseline and after three months. In this case, the weight and height were self-reported. The diet of the volunteers was analyzed from the app data at 1, 2, and 3 months.

### 2.3. Ethical Considerations

The Andalusian Regional Government’s Biomedical Research Ethics Committee approved the study protocol for both trials. In addition, the volunteers signed an informed consent form prior to their inclusion in each of the studies. All personal data obtained in this study were confidential and treated in accordance with the Organic Law on Personal Data Protection and Digital Rights Guarantee [44].

### 2.4. Statistical Analysis

The sample size was estimated for the main variables of the study. The recruitment of 104 participants for the randomized control trial, 55 participants in the intervention group and 49 in the control group, and 40 participants for the online trial was considered sufficient to detect a significant difference of 10% in the improvement of the adherence to the Mediterranean diet, with an alpha risk level of 0.05, a standard deviation of 10%, and a statistical power of 99%. This sample size is above the minimum recommended for a pilot study (*n* = 12) [45].

The results were expressed as the mean ± standard deviation for quantitative variables, with the frequency distribution being used for qualitative variables. The results were analyzed by intention-to-treat, including all the patients in the groups. Student’s *t*-test was applied to compare the means between the two groups. The paired *t*-test was used to assess changes within the same group. In order to determine the impact of the intervention, we compared the changes observed in the different weeks between the CG and IG by the ANCOVA test, adjusting for baseline measurements of each variable. A value of *p* < 0.05 was considered statistically significant. IBM SPSS software for Windows version 26.0 was used for statistical data analyses.

## 3. Results

### 3.1. Primary Care Counseling plus App: Randomized Control Trial

#### 3.1.1. Participants’ Baseline Characteristics 

The study included 104 participants distributed in either the IG (*n* = 55) or the CG (*n* = 49). Approximately one-quarter of the sample was men (25.5% in the IG and 22.5% in the CG). The average age was 58.5 ± 9.1 and 53.4 ± 15.8 years and the average BMI was 33.0 ± 7.9 and 31.0 ± 6.3 for the IG and CG, respectively. No significant differences were observed in the demographic and baseline characteristics between the two groups (Table 1).

At baseline, the mean percentage intakes of carbohydrates, proteins, total fats, and fiber did not meet the WHO and EFSA dietary reference values [46,47,48,49] in either the intervention or the control group (Table 2). However, the mean percentage of free sugar intake followed the WHO recommendations of less than 5% of the total energy for both groups (Table 2). 

#### 3.1.2. Impact of SAlBi educa on Energy and Macronutrients Intake, Adherence to MD, and Nutritional Knowledge

In the CG, which received counseling only, no significant differences were detected in the carbohydrate, proteins, total fats, fiber, and free sugar intake over one month (Table 2). However, members of the IG (counseling + app) significantly increased carbohydrates and fiber intake, while total fats intake between baseline and week 4 decreased (Table 2). An increase in fruit and vegetable intake was also observed at week 4, although with no significant differences. A decreasing trend in the protein intake was detected over the month, although without any significant differences. Total energy and free sugar intake did not significantly change in the intervention group over the month.

Table 3 shows the impact of SAlBi educa on changes in macronutrient intake over one month. Adjustments for each variable’s baseline measures were considered in order to counteract differences between the CG and IG for the energy, nutrients, and food group intakes at baseline. A significant increase was observed in the IG with respect to the CG in carbohydrate intake at week 4 (7.7% (95% CI: 0.16 to 15.2). Additionally, a significant effect of SAlBi educa was detected for total fat intake, which decreased with respect to the control group at week 4 (−5.7% (95% CI: −10.4 to −1.15)). A beneficial decreasing trend was also observed in the IG with respect to the CG for the protein intake percentage (−2.2% (95% CI: −4.8 to 0.5)), although it was not significant (Table 3). No significant differences in free sugar intake percentages were detected in the IG with respect to the CG.

With regard to adherence to the Mediterranean diet, significant differences were only observed in the CG mean score at week 4 (Table 2). No significant differences were observed in the mean differences between IG and CG (Table 3). However, 46.7% (26/55) and 40.6% (20/49) of participants in the IG and CG, respectively, improved the MedDiet adherence score at week 4. The mean punctuation increase was 2.3 ± 1.3 and 2.6 ± 1.8 for IG and CG, respectively, corresponding to an improvement percentage in adherence to the Mediterranean diet of 33.5 ± 22.2% and 44.5 ± 45.1% for IG and CG, respectively.

A significant increase was detected in the CG for the GNKQ mean scores only (Table 2). A total of 65.6% of participants (32/49) improved their score, with the mean punctuation increase being 6.6 ± 4.0. Additionally, 60% of participants (33/55) in the IG improved GNKQ scores with a mean punctuation increase of 7.0 ± 7.7. However, no significant differences were found in the mean differences between IG and CG (Table 3). The significant increase in the GNKQ and MedDiet scores in the CG reinforces the efficiency of dietary counseling in primary healthcare. 

A total of 60% (33/55) and 72.7% (36/49) of participants lost weight in the IG and CG, respectively. The mean weight loss was −1.1 ± 1.7 kg and −1.0 ± 1.4 kg for IG and CG, respectively, with no significant differences between them (Table 3). BMI did not show significant differences in either the CG or the IG (Table 2 and Table 3).

### 3.2. Impact of SAlBi Educa on Healthy Eating Habits during the COVID-19 Pandemic: Online Trial Study

#### 3.2.1. Baseline Characteristics of Participants

Of the individuals included in the study, 67.5% were women; the mean age was 43.1 ± 12.5 years, and the mean BMI was 26.9 ± 5.1 (Table 1). Most of the participants possessed university studies (87.5%; 35/40) and were employed (67.5%, 27/40). At baseline, the mean intake of carbohydrates, proteins, total fats, fiber, and free sugar were not aligned with the dietary reference values established by the WHO and the EFSA [46,47,48,49].

#### 3.2.2. Effect of SAlBi Educa over Three Months

Significant differences were found in carbohydrate, total fats, fruit and vegetables, legumes, starchy foods, red meat, and processed meat intakes over three months (Table 4). Carbohydrate intake significantly increased from month 1 (3.5% (95% CI: −1.0 to 5.8)) and was maintained at months 2 and 3. Moreover, fruit and vegetable intake was also significantly enhanced from the first month (211.6 g/day (95% CI: 126.0 to 296.4)). This also increased in months 2 (289.0 g/day (95% CI: 178.9 to 399.1)) and 3 (266.3 g/day (95% CI: 130.0 to 402.6)). Additionally, legumes intake significantly increased at months 2 and 3 (7.3 g/day (95% CI: 0.3 to 14.4) and 7.7g/day (95% CI: 0.2 to 15.1), respectively). Moreover, starchy food intake (cereals, including whole grains and cereal-derived products, such as bread, pasta, rice, and couscous; and potatoes) was significantly enhanced at months 1, 2, and 3 (23.5 g/day (95% CI: 2.1 to 44.9), 34.9 g/day (95% CI: 1.9 to 67.8), and 36.4 g/day (95% CI: 1.1 to 71.7), respectively), while total fat intake significantly decreased from month 1 (−4.1% (95% CI: −6.6 to −1.5)), and was still decreasing at months 2 (−5.3% (95% CI: −8.0 to −2.7)) and 3 (−5.9% (95% CI: −8.9 to −3.0)). Furthermore, red meat intake decreased significantly at month 3 (−17.5 g/day (95% CI: −34.0 to −1.1)) and processed meat at month 2 (−6.6 g/day (95% CI: −13.1 to −0.1)). Although fiber and wholemeal bread intake showed an increasing trend over the three months, no significant differences were found (Table 4). Proteins and free sugar intake did not present significant differences between baseline and months 1, 2, and 3, either. 

The MedDiet adherence score showed significant differences at month 3 (Table 4). A total of 57% of participants (23/40) improved their score with a mean punctuation increase of 2.3 ± 1.2. No significant differences were observed for GNKQ scores at 3 months. However, 30% of participants (12/40) increased their scores, with a mean punctuation increase of 4.4 ± 2.2. A total of 65% (26/40) of participants lost weight at three months. The mean weight loss was −0.8 ± 2.1 kg. However, BMI did not show any significant differences at three months (Table 4).

## 4. Discussion

SAlbi educa is a nutrition app, which provides tailored recommendations in order to balance the user’s diet. SAlbi educa’s usability in a real environment has been proven [25]. Additionally, the present study shows that SAlBi educa is a helpful tool for dietary counseling in primary healthcare for improving dietary habits. Its major contribution consisted of a significant increase in the carbohydrate intake percentage (7.7%) and a significant decrease in the intake percentage of total fat (5.7%). Moreover, our results also demonstrate that SAlBi educa itself was able to improve dietary habits significantly (carbohydrate, total fat, fruit and vegetables, legumes, starchy foods, red meat, and processed meat) of a population during the COVID-19 pandemic when health professionals found themselves forced to concentrate their efforts on other priorities. 

At baseline, carbohydrate, protein, total fat, and fiber mean intakes of participants in both studies did not meet WHO and EFSA dietary recommendations [46,47,48,49] (Table 2 and Table 4). These findings agree well with the observed dietary intake of the Spanish population as a whole, which, over the last 50 years, has deviated significantly from the traditional Mediterranean diet pattern [30,31]. Interestingly, not only did SalBi educa significantly increase carbohydrate intake and decrease the total fat intake but, by the end of the studies (Table 2 and Table 4), it had also aligned the mean values for both macronutrients with the WHO and EFSA dietary reference values for a healthy diet [46,47,48,49]. Since the free sugar intake did not change over the study, the observed increase in carbohydrates appears to result, in part, from an increase in fruit and vegetable consumption, although with no significant differences between CG and IG. This result is supported by a significant increase in fiber intake in the IG only at 4 weeks. This may also suggest that other fiber-rich food groups, such as legumes and whole grains, might be contributing to the significant enhancement of carbohydrates.

A recent systematic review shows that most studies on nutritional eHealth interventions focus on weight loss, while only a few examine the impacts of changes in macronutrient intake [50]. SAlBi educa results agree well with the EVIDENT II nutrition app [24], which increased the intake percentage of carbohydrates (1.1%) and decreased the intake percentage of total fats (1.0%) in the general population. Additionally, the EVIDENT 3 app also enhanced the carbohydrate intake (plus 3.8 g/day) and decreased the total fat intake (minus 4.7 g/day) in an overweight and obese population, although the app’s effect was not significant [51]. In the same sense, the Lost it! app showed a reduction in total fat intake (−4.9%) in an obese population at 6 months, although with no significant differences [52]. It should be noted that the effects of the abovementioned studies were reported after 3, 6, and 12 months. However, SAlBi educa’s effect was noticeable after only one month of the intervention, and the online three-month study supported the notion that its effect on carbohydrate and fat intake is maintained and even improves after three months. 

An increase in fruit and vegetable intake has been associated with risk reductions for quite a number of non-communicable diseases, such as obesity, cardiovascular diseases, and type II diabetes, among others [53,54]. The WHO recommends a daily intake of at least 400 g of fruit and vegetables, which corresponds to five servings of 80 g per day [2]. SalBi educa has shown a significant enhancement in fruit and vegetables intake in the online 3 months study from baseline to 1, 2, and 3 months (mean differences: 211.6 g/day (2.6 servings), 289.0 g/day (3.6 servings), and 266.3 g/day (3.3 servings), respectively). In these cases, SAlbi educa managed to align the mean intake (Table 4) with the WHO daily intake recommendation. A recent systematic review has shown that certain apps are able to increase the daily intake of fruit and vegetables [55]. The ‛Make Better Choices 2′ app significantly increased fruit and vegetable intake by 1.74 servings at 3 months compared to the control group in an adult population [22]. Additionally, the ‛Vegethon’ app, aimed at promoting fruit and vegetable intake, has been reported to significantly enhance vegetable consumption by one serving/day (mean differences between intervention and control group) at two months in an overweight adult population [23]. Although SalBi educa is not exclusively focused on promoting fruit and vegetable consumption, its effect is within the range of apps targeting this food group.

Scientific evidence supports the notion that obesity and its associated NCDs are related to a higher risk of suffering COVID-19 and its effects [56]. The potential mechanism appears to be related to the enhancement of the angiotensin-converting enzyme 2 (which the virus uses to enter the cells) in the adipose tissue of obese people, the chronic pro-inflammatory state of subjects with obesity, and thoracic expansion limitation. This results in suppression in the pulmonary parenchyma at the lung bases due to the central adiposity, among others [57,58]. Therefore, the efforts to prevent obesity would also limit this infectious disease, its conditions, and especially its severity.

The COVID-19 pandemic modified both dietary habits and the traditional dietary counseling protocol. A recent systematic review shows that not only was there an increase in the intake of fruit and vegetables, legumes, cereals and olive oil, but also in dairy, sandwiches, and sweets, and a decrease in fish consumption [59]. Additionally, the total food intake and the number of meals and snacks also increased [32,33,34,35,36,37,38]. The Mediterranean diet might represent a potential strategic approach to tackle both short- and long-term conditions associated with the COVID-19 infections and severity, as well as improving the wellbeing of the population affected [60]. 

The present study has shown that SalBi educa, which offers personalized dietary counseling based on the Mediterranean diet model, improved the dietary profile in an adult population during the COVID-19 pandemic. It significantly increased the carbohydrate intake (from fruits and vegetables, legumes, and starchy foods, which are rich in complex carbohydrates) and decreased the total fat intake, due to a reduction in the consumption of red and processed meat, both of which are rich in saturated fat. Several studies have shown that high red and processed meat consumption can increase the risk of colorectal cancer, heart disease, diabetes, and other chronic diseases [61,62]. Thus, an optimally healthy diet would moderate the consumption of red and processed meats [63]. Therefore, the reduction in fat intake, from foods rich in saturated fat, accompanied by an increase in the consumption of complex carbohydrates, supports SalBi educa’s impact on changing dietary intake in order to follow a healthy diet. Additionally, SalBi educa significantly enhanced the participants’ adherence to the Mediterranean diet at three months (MedDiet score: 9.6 ± 1.9). 

Traditionally, dietary counseling in primary healthcare in the Andalusian region consisted of group sessions or individual visits including recommendations to follow a healthy diet, and printed leaflets [3]. However, dietary counseling has been reduced and group visits have not been possible since the COVID-19 pandemic due to several limitations, such as capacity and health professionals’ limitations, who have found themselves forced to concentrate their efforts on other priorities. Therefore, SalBi educa has proven to be a useful supplementary tool for the nutritional counseling given by health professionals, improving the efficiency of this particular aspect of public health.

The present studies present several limitations. First, and with the exception of height and weight measurements in the randomized control trial, all of the data collected in this study were self-administrated by the participants. There could, therefore, have been social approval bias, which might underestimate genuine dietary intake. Further improvement of the app should include the automatic gathering of physical activity recorded by an activity tracker wristband for a more precise measurement of this component of energy expenditure. The nature of the present studies made it impossible to blind the participants. Although using another nutrition app was an exclusion criterion, we cannot guarantee that they did not use one during the study periods. 

The present studies have shown the effectiveness of SalBi educa over one- and three-month periods. In a previous study, we evaluated adherence to SalBi educa for a one-month period, being 81.3% (participants who were tracking at least 3 days/week with two main eating occasions) [25]. Several studies have shown that nutrition apps present similar adherence at 6 and 11 weeks [64,65]. Therefore, a follow-up study at 6 and 12 months may be convenient to evaluate whether the changes in the dietary profile have also resulted in a significant loss of body weight and BMI [66].

Both RCT and online trials recruited volunteers through the four participating healthcare centers. Additionally, the online trial was also open to people not undergoing primary healthcare dietary counseling. Both trials were open to the general population with no restriction on educational level, socioeconomic status, sex, age, or BMI. Although the characteristics of the RCT population in both CG and IG did not significantly differ, differences between the population in the RCT and online trial were observed. The online trial presented a higher percentage of participants in employment with a higher educational level, and a lower mean BMI. Despite these differences, the main results were highly similar in both trials, reinforcing the potential of the application’s use in different scenarios/populations. In order to evaluate whether using SAlBi educa is also efficient for other populations, further studies should be conducted to focus on specific target populations, such as those with a low educational level, a low socioeconomic status, an obese population, and different ages. Additionally, a larger sample population in future studies would be convenient. Moreover, the inclusion of critical micronutrients (iron, folate, and vitamins B12 and A) [67] should be considered for further research directions. 

## 5. Conclusions

The present study showed that SAlBi educa proved to be useful for significantly increasing carbohydrate intake and decreasing total fat intake. Additionally, the MedDiet adherence score was significantly enhanced at three months. The SAlBi educa nutrition app has demonstrated its potential in persuading users to improve their diet, based on the Mediterranean model, and its potential to become an effective solution for supporting Nutrition Counseling in primary healthcare, including in special situations such as during the COVID-19 pandemic. 

## Figures and Tables

**Table 1:** Characteristics of the study populations at baseline.

Baseline Characteristics	Randomized Control Trial			Online Trial
	IG (Counseling + App) (*n* = 55)	CG (Counseling Only) (*n* = 49)	*p*-Value	OG (App Only) (*n* = 40)
	Mean ± SD	Mean ± SD		Mean ± SD
Age, years	58.5 ± 9.1	53.4 ± 15.8	0.207	43.1 ± 12.5
Sex, *n* (%)				
Women	41 (74.5)	38 (77.5)	0.720	27 (67.5)
Men	14 (25.5)	11 (22.5)		13 (32.5)
Educational level, *n* (%)				
Elementary school	21 (38.2)	17 (34.7)	0.720	2 (5)
High school	20 (36.4)	16 (32.6)		3 (7.5)
University studies	14 (25.4)	16 (32.6)		35 (87.5)
Work situation, *n* (%)				
Employed	18 (32.7)	14 (28.5)	0.591	27 (67.5)
Unemployed	13 (23.7)	16 (32.6)		11 (27.5)
Retired	24 (43.6)	19 (38.7)		2 (5)
BMI (kg/m^2^)	33.0 ± 7.9	31.0 ± 6.3	0.280	26.9 ± 5.1

IG: Intervention group; CG: Control group; OG: Online group; SD: Standard deviation; BMI: Body mass index.

**Table 2:** Energy and macronutrients intake, adherence to MD, and nutritional data over a month.

	IG (Counseling + App)				CG (Counseling Only)			
	W1 Baseline Mean ± SD	W2 Mean ± SD	W3 Mean ± SD	W4 Mean ± SD	W1 Baseline Mean ± SD	W2 Mean ± SD	W3 Mean ± SD	W4 Mean ± SD
Energy (Kcal/day)	1210.9 ± 290.9	1282.3 ± 368.2	1314.1 ± 354.6	1330 ± 319.5	1672.9 ± 427.6	1539.7 ± 359.3	1553.8 ± 381.5	1463.7 ± 264.5
Carbohydrates (% of energy)	37.9 ± 6.0 ^a^	42.3 ± 6.2	44.2 ± 9.3	46.4 ± 7.8 ^b^	37.5 ± 7.8	38.3 ± 6.3	37.9 ± 5.6	37.2 ± 7.8
Proteins (% of energy)	19.0 ± 4.4	17.3 ± 2.5	17.2 ± 4.4	16.4 ± 3.0	18.4 ± 5.1	18.3 ± 3.8	18.0 ± 3.9	18.8 ± 3.9
Total fats (% of energy)	41.4 ± 5.7 ^a^	38.6 ± 7.5	36.4 ± 5.8	33.7 ± 5.8 ^b^	42.4 ± 6.7	42.0 ± 6.3	41.4 ± 4.4	42.9 ± 4.8
Fiber (g/day)	13.5 ± 6.2 ^a^	15.2 ± 8.0	16.8 ± 7.7	19.1 ± 7.3 ^b^	18.3 ± 8.1	18.2 ± 6.9	19.5 ± 7.7	17.3 ± 6.0
Free sugar (% of energy)	4.7 ± 2.8	4.5 ± 2.8	5.1 ± 3.1	4.0 ± 2.5	4.0 ± 2.6	3.8 ± 2.4	4.1 ± 2.8	3.6 ± 1.9
Fruit and vegetables (g/day)	364.7 ± 198.1	384.5 ± 229.6	373.1 ± 197.8	562.8 ± 309.0	598.7 ± 321.4	569.2 ± 186.7	610.7 ± 272.0	545.0 ± 218.2
MedDiet adherence score	8.7 ± 2.2			9.1 ± 2.0	8.2 ± 2.1 ^a^			9.7 ± 1.9 ^b^
GNKQ score	34.8 ± 8.8			36.2 ± 7.5	33.5 ± 9.0 ^a^			38.2 ± 5.2 ^b^
Weight (kg)	85.4 ± 22.0			84.4 ± 21.7	85.3 ± 18.5			84.3 ± 18.0
BMI (kg/m^2^)	33.0 ± 7.9			31.9 ± 8.0	31.0 ± 6.3			31.3 ± 6.7

^a^ and ^b^ mean *p* < 0.05 within the same nutrient, and questionnaire scores from baseline to week 4.

**Table 3:** SAlBi educa’s impact in terms of changes in macronutrient intake, adherence to MD, and nutritional knowledge over one month.

	Mean Difference (IG–CG) Week 2		Mean Difference (IG–CG) Week 3		Mean Difference (IG–CG) Week 4	
	Mean Difference (95% CI)	*p*-Value	Mean Difference (95% CI)	*p*-Value	Mean Difference (95% CI)	*p*-Value
Energy (Kcal/day)	−127.9 (−38.3 to 293.9)	0.126	−32.5 (−244.5 to −179.4)	0.757	136.4 (−98.6 to 371.4)	0.238
Carbohydrates (% of energy)	3.6 (−0.4 to 7.8)	0.078	5.1 (−0.5 to 10.8)	0.076	7.7 (0.16 to 15.2) *	0.045
Proteins (% of energy)	−0.9 (−3.3 to 1.4)	0.417	−1.2 (−3.9 to 1.3)	0.330	−1.1 (−5.1 to 2.9)	0.589
Total fats (% of energy)	−1.6 (−5.9 to 2.5)	0.421	−3.3 (−7.5 to −0.8)	0.118	−5.7 (−10.4 to −1.15) *	0.016
Fiber (g/day)	1.4 (−2.6 to 5.4)	0.495	0.5 (−3.3 to 4.5)	0.771	1.5 (−6.4 to 9.5)	0.689
Free sugar (% of energy)	−0.1 (−1.6 to 1.4)	0.876	0.5 (−1.4 to 1.4)	0.594	0.6 (−2.0 to 3.3)	0.624
Fruit and vegetables (g/day)	73.2 (−87.3 to 233.8)	0.359	−43.3 (−121.6 to 128.0)	0.615	140.2 (−956. to 376.1)	0.226
MedDiet adherence score					−1.3 (−3.3 to 0.6)	0.180
GNKQ score					−0.9 (−4.9 to 2.9)	0.624
Weight (kg)					−0.1 (−0.9 to 0.7)	0.788
BMI (kg/m^2^)					−0.2 (−0.6 to 0.1)	0.109

* *p* < 0.05.

**Table 4:** Energy and macronutrient intake, adherence to MD, and nutritional knowledge scores over three months in the online intervention.

Nutrients	Online Intervention (App Only)			
	Baseline Mean ± SD	Month 1 Mean ± SD	Month 2 Mean ± SD	Month 3 Mean ± SD
Energy (Kcal/day)	1531.5 ± 365.3	1480.7 ± 341.4	1409.7 ± 378.6	1409.7 ± 378.6
Carbohydrates (% of energy)	39.1 ± 6.2	43.9 ± 7.2 **	43.9 ± 7.2 ***	43.9 ± 7.6 **
Proteins (% of energy)	17.4 ± 3.1	17.7 ± 3.6	17.7 ± 3.3	16.8 ± 3.3
Total fats (% of energy)	40.7 ± 6.0	36.5 ± 7.2 **	35.3 ± 7.6 ***	34.7 ± 8.8 ***
Fiber (g/day)	15.5 ± 5.9	17.9 ± 8.4	18.5 ± 9.2	18.8 ± 7.8
Free sugar (% of energy)	6.3 ± 3.3	5.7 ± 3.5	5.7 ± 3.1	5.7 ± 3.1
Fruit and vegetables (g/day)	278.4 ± 147.3	489.67 ± 265.74 ***	567.44 ± 320.86 ***	626.89 ± 216.99 ***
Legumes (g/day)	13.7 ± 14.6	19.0 ± 9.5	21.9 ± 13.7 *	21.5 ± 8.8 *
Starchy food (g/day)	120.6 ± 41.8	144.2 ± 45.2 *	155.5 ± 76.6 *	157.8 ± 76.1 *
Wholemeal bread (g/day)	27.3 ± 35.0	35.4 ± 29.5	39.9 ± 31.6	52.3 ± 45.6
Red meat (g/day)	50.1 ± 30.9	36.2 ± 37.8	32.4 ± 23.4	28.8 ± 21.6 *
Processed meat (g/day)	18.2 ± 7.9	12.6 ± 9.1	11.4 ± 6.0 *	12.1 ± 11.0
ADM score (mean)	8.2 ± 2.0			9.6 ± 1.9 *
GNKQ score (mean)	34.3 ± 6.7			36.5 ± 5.9
BMI (kg/m^2^)	26.9 ± 5.1			26.2 ± 6.3

* *p* < 0.05; ** *p* < 0.01; *** *p* <0.001 between baseline and month 1, month 2, and month 3.

## Data Availability

The datasets used and/or analyzed during the current study are available from the corresponding author on reasonable request.

## Supplementary Materials

The following supporting information can be downloaded at: https://www.mdpi.com/article/10.3390/nu14102061/s1, Figure S1: Description of the dietary counseling sessions.
