# Supplementary material for: Short-Term Pilot Study to Evaluate the Impact of Salbi Educa Nutrition App in Macronutrients Intake and Adherence to the Mediterranean Diet: Randomized Controlled Trial
Source: Nutrients. 2022 May 14;14(10):2061. doi: 10.3390/nu14102061 (PMC9146242; doi:10.3390/nu14102061)
Supplement: Supplementary file 1 [file nutrients-14-02061-s001.zip › nutrients-1697014-supplementary.pdf]

COUNSELING SESSIONS

SAIBi educa

SESSION 1. BASIC CONCEPTS. MEDITERRANEAN DIET.

- Take anthropometric measurements (weight and height)
- Presentation (10´)
  - Presentation of the Healthcare Professional and Workshops
  - Presentation of the participants
- Workshop presentation (40')
- Delivery of dietary record (CG only)

SESSION 2. NUTRIENTS AND FOOD

- Collection of the dietary records (CG only)
- Questions and doubts raised during the week (20´)
- Explain the workshop (30´): Establish a dialogue to find out if they have understood the key concepts
- Group activity (20'): Portions of habitual consumption. Activity with food and scales
- Delivery of dietary record (CG only)

SESSION 3. PHYSICAL ACTIVITY

- Collection of the dietary records (CG only)
- Questions and doubts raised during the week (20´)
- Oral presentation on some key ideas of physical activity and rest (15´)
  - Not being in front of TV for more than two hours in a row
  - At least 150–300 minutes throughout the week of moderate-intensity aerobic physical activity (PA) are recommended
  - Or at least 75–150 minutes of vigorous-intensity aerobic physical activity; or an equivalent combination of moderate- and vigorous-intensity activity throughout the week
- Group activity (30')
  - Demonstrate the physical activities to be carried out: Divide the group into 2-3 groups
  - Finally, specify the time dedicated to various activities and rest.
- Ask them to bring packages to the next session to discuss the labels
- Delivery of dietary record (CG only), Mediterranean Diet adherence, and GNKQ questionnaires

SESSION 4. PURCHASING FOOD AND UNDERSTANDING LABELING

- Collection of dietary record (CG only), Mediterranean Diet adherence, and GNKQ questionnaires
- Take the anthropometric measurements (weight)
- Questions and doubts raised during the week (20´)
- Oral presentation on how to make a healthy purchase (20´)
  - What to buy for healthy eating
  - The buying cycle: before, during and after
  - Sustainability and environment
- Group activity (40')
  - Label analysis
- Establish a dialogue to find out if they have understood the key concepts and difficulties

Figure S1. Description of the dietary counseling sessions.
